# Investigating foliar application of bulk and nanoparticles titanium dioxide on fennel productivity to mitigate the negative effects of saline irrigation water

**DOI:** 10.1186/s12870-024-04996-8

**Published:** 2024-04-23

**Authors:** Aisha M. A. Ahmed, Khalid A. Khalid, Faten S. A. Zaki

**Affiliations:** 1 Botany Department, National Research Centre, El Buhouth St., Cairo, 12622 Dokki Egypt; 2 Medicinal and Aromatic Plants Department, National Research Centre, El Buhouth St., Cairo, 12622 Dokki Egypt

**Keywords:** Fennel, Irrigation water, Ti forms, Essential oil, Chemical fractions

## Abstract

**Background:**

Fennel essential oils are fragrance compounds used in food and pharmaceutical sectors. One of the major impediments to expansion of fennel farming in Egypt's reclamation areas is saline water. Titanium dioxide (TiO_2_) or TiO_2_ nano particles (TiO_2_NP) can be utilized to boost the yield of aromatic plants cultivated under saline irrigation water. Saline water, particularly which contains sodium chloride can harm fennel plant; consequently, it was predicted that fennel production would fail in Egypt's reclaimed area, where the primary source of irrigation is groundwater consisting sodium chloride. This study sought to help fennel respond to sodium chloride by applying Ti forms to their leaves in order to reduce the detrimental effects of sodium chloride on them for expanding their production in the newly reclamation areas as a natural source of essential oil. Ti forms were applied as foliar application at 0, 0.1, 0.2 TiO_2_, 0.1 TiO_2_NP, and 0.2 TiO_2_NP, mM under irrigation with fresh water (0.4 dS m^−1^), or saline water (51.3 mM or 4.7 dS m^−1^).

**Results:**

Plants exposed to 0.1 mM TiO_2_NP under fresh water resulted in the maximum values of morphological characters, estragole, oxygenated monoterpenes and photosynthetic pigments; while those subjected to 0.1 mM TiO_2_NP under saline water gave the greatest values of essential oil, proline, antioxidant enzymes and phenols. The greatest amounts of soluble sugars were recorded with 0.2 mM TiO_2_NP irrigated with saline water. Plants subjected to 0 mM TiO_2_ under saline water produced the greatest values of flavonoids, hydrogen peroxide and malondialdehyde.

**Conclusion:**

To mitigate the negative effects of salty irrigation water on fennel plant production, TiO_2_NP application is suggested as a potential strategy.

## Introduction

Herbs have undergone substantial research due to the growing demand for their usage in different applications, including food, medicine and pharmaceuticals. Herbs provide a variety of byproducts that are economically valuable for raising the country's income [1]. The most vital active components of aromatic herbs are essential oils (EOs) [2]; they can be used in numerous industries including medicines, cosmetics, perfumes, fragrances, flavors, food additives, agrochemicals and biopesticides [3]. According to scientific research, utilizing EOs from aromatic herbs is preferable than using synthetic chemicals for a number of reasons because the latter have harmful side effects, particularly carcinogenic ones [3]. Fennel (*Foeniculum vulgare* Mill) is a medicinal and spice herb belonging to Apiaceae family [4]; its EO is used in food, aromatherapy and pharmaceutical industries as flavoring and fragrance ingredients [5].

Increasing population has caused serious problems in today’s societies due to the lack of suitable regions for agriculture; governments frequently increase the cultivation of desert lands, which are frequently found in arid or semi-arid regions [6]. Water shortage is a global issue that is getting worse in arid and semi-arid regions; poor quality water, including salty groundwater is frequently utilized to make up for this deficit [7]. Using saline groundwater, which normally contains solutes in different amounts significantly, alters soil properties. With use of salt water for irrigation, soil becomes more salinized and salt deposits form in the root zone, which causes poor plant development and degradation [8]. Where, high amounts of salted irrigation water (SALIW) can lead to sodium and reactive oxygen species (ROS) buildup in plant tissues, decreases in basic elements and water uptakes causing oxidative and ionic stress, imbalance in nutrients, injuries to cell membranes and a drop in photosynthetic pigments (POTSP), which impedes development and growth [9]. On the other hand, SALIW is crucial for the production of proteins (PROTs), accumulation of total phenols (TPHEN) and total flavonoids (TFLAV), lipid metabolism, generation of carbohydrates (CARB) and a variety of natural product metabolites, particularly the composition of EOs in aromatic plants [10–13]. Impact of SALIW treatments on the equilibrium and uptake of essential macro- and micronutrients; this lowers protein and chlorophyll levels, which in turn cause reductions in growth and dry matter values [10–13]. Due to its ability to scavenge ROS, store CARB, and provide osmo-protection, CARB may be the primary factor contributing to the increase in CARB under SALIW [12.13]. In response to SALIW, plants produce more TPHEN, TFLAV, and EO, which is in line with their function as antioxidants that reduce oxidative damage [10–13]. The effects of SALIW vary depending on plant species [13, 14]. SALIW with sodium chloride (NaCl) in fennel leads to decreased plant growth, fresh and fruit yield, relative water content, EO, lipids, oleic and linoleic acids, POTSP, potassium (K), calcium (Ca) and magnesium (Mg); while, proline (PROL), CARB, TPHEN, malondialdehyde (MDA), hydrogen peroxide (H_2_O_2_), antioxidant enzymes activity (AOEA), total soluble solids (TSS), sodium (Na), major components of EO (limonene, fenchone, estragol, *trans*-anethol) and palmitic acid increased [15–21].

Scientific studies have been carried out to examine a number of strategies to increase the output of aromatic plants under stressful situations. Titanium (Ti) as titanium dioxide (TiO_2_) or TiO_2_ Nano particles (TiO_2_NP) can be used to increase the output of aromatic herbs grown under salt stress [22]. Ti is a favorable element found in the majority of rocks, sediments and sands [23], it is the tenth most prevalent element in the Earth's crust; furthermore, it is second transition metal [23]. Ti has a great affinity for oxygen, which it can be found naturally as TiO_2_ [24]. TiO_2_NP may be produced naturally and is utilized in a variety of industrial applications [24]. Low concentrations of Ti applied through the roots or leaves have been reported to enhance crop performance by promoting the activity of specific enzymes, increasing chlorophyll content and photosynthesis delaying in chloroplast senescence, promoting nutrient uptake, enhancing stress tolerance and improve the plant production [25]. Ti forms (TiO_2_ or TiO_2_NP) had a favorable impact on plant development, antioxidant capacity, AOEA activities, total soluble sugars (TSOLS), amino acids, EOs and their major constituents, pH value, titratable acidity (TA), electrical conductivity (EC) and TSS; as well as a decrease in H_2_O_2_ and MDA contents in plants grown in SALIW [26–28].

Egyptian government is working to increase the production of medicinal and aromatic plants that are grown in desert areas. This is in the context of the government's dedication to strengthening pharmaceutical economics and supporting the pharmaceutical sectors in Egypt. Unfortunately, those areas have only groundwater that contains salts, especially NaCl. Previous research has shown that fennel plant can tolerate small amounts of SALIW, but that excessive salt concentration, especially those containing NaCl can injure fennel plant; prior research has demonstrated that fennel plants can withstand saline levels of up to 1.6 dS m^−1^; above that point, the plant's output and active ingredient concentration start to decline [21]. Consequently, this research aimed to lessen the negative impacts of SALIW on fennel plants by foliar spraying fennel plants with Ti forms (TiO_2_ or TiO_2_NP) to help them become tolerant to SALIW; this research has never been done previously, particularly in Egypt.

## Materials and methods

### TiO_2_ and TiO_2_ NPs characterization

**Fig. 1:**
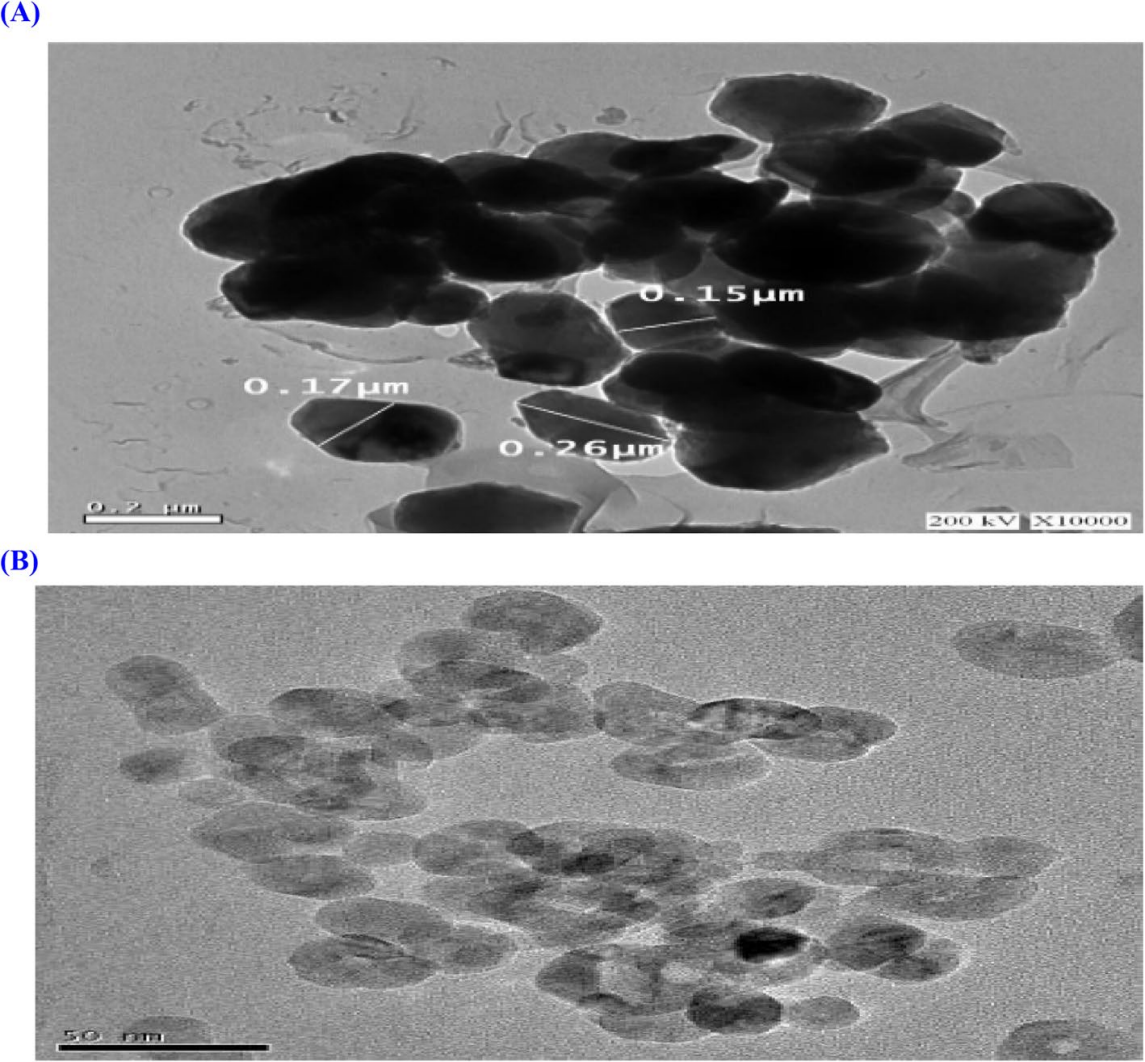
**A** Transmission electron microscope** (**TEM) image of titanium dioxide (TiO_2_). **B** TEM image of titanium dioxide nano particles (TiO_2_NPs)

Morphological characterization of TiO_2_ and TiO_2_NPs was carried out using TEM (Fig. 1A and B). TEM showed that TiO_2_ shape was semi-round and platy with average particle size 1933 nm (1.9 µm). DLS of TiO_2_ showed the size of particulate matter was about 1800 nm. The zeta potential of TiO_2_ in aqueous solution was repossessed which was 16 mV. TEM presented that TiO_2_ NPs were in definite particles with tetragonal shape at resolution scale 50 nm and approved by XRD. It is also clear from Fig. 1B that NPs are in dispersed as well as in aggregated form and the average size was 21 nm. DLS is used to determine the size of NPs which shows the size of particulate matter was about 19–20 nm. The zeta potential of TiO_2_ NPs in aqueous solution was rescued which was16.1 mV.

### Experimental techniques

Two seasons (2021/2022 and 2022/2023) of experiments were conducted in a greenhouse at the National Research Centre in Giza, Egypt, with parameters set to 22/14 °C, 52/31% RH day/night and light intensity: 3700 lx. Fennel seeds were purchased from the Department of Medicinal and Aromatic Plants at Ministry of Agriculture in Giza, Egypt. In plastic pots (30 cm diameter and 50 cm height), seeds were planted in the second week of October during both seasons; there were 10 kg of air-dried soil in each pot. Six weeks after planting, seedlings were thinning to three plants per pot. Pots were separated into two main groups. The first one was subjected to various concentrations of TiO_2_ forms at 0, 0.1, 0.2, 0.1NP, and 0.2NP mM with fresh irrigation water (FRIW; 0.4 dS m^−1^). The second group received the same TiO_2_ treatments as the first, but it received 51.3 mM SALIW. Fennel plants were exposed to NaCl concentration after 45 days from planting (during vegetative stage); selection age was used according to Schreiber [29] and Day [30]. To make irrigation water highly soluble NaCl salt was employed. Salt concentration was measured by adding the proper quantity of NaCl to water and adjusting it using a portable EC meter; irrigation carried out according to field water capacity (FWC) which scored 60%. At every seven days, the soils found in pots of the second group were leached by tap water as a consequence of the salinity check; if there was no leaching when using SALIW, it can cause salt buildup in pots. TiO_2_ (≥ 99%) and TiO_2_NP (Nano powder 21 nm, particle size, TEAM, ≥ 99.5% trace metal basis, Sigma Aldrich. Sigma Aldrich Company supplied bulk anatase TiO_2_ particles with 99% purity TiO_2_NPs (https://www.sigmaaldrich.com/EG/en/product/aldrich/637254) with particle size 21 nm and purity more than 99%.The structural properties of TiO_2_and TiO_2_ NPs were determined by Transmission Electron Microscope (TEM) model type of (JEOL-JEM-2100). TEM was used for morphological characterization. For dynamic light scattering (DLS) and zeta potential, aqueous solution of desired concentration of TiO_2_ and TiO_2_NPs (1 g L^−1^) was prepared by dissolving TiO_2_ and TiO_2_NPs in double de-ionized water (DDW). The solution was sonicated in a bath sonicator (~ 40 W) for 30 min. The particle size of the prepared TiO_2_ and TiO_2_NPs and zeta potential in an aqueous suspension were determined by measuring DLS with a Zetasizer (Malvern, UK).Due to selection of anatase type of bulk and nano titanium samples, X-Ray Diffraction (XRD) was not employed, since certified products were used with known particle shape. XRD measurement revealed that anatase level of the bulk TiO_2_ particles are crystalline in nature and TiO_2_ NPs were tetragonal particles [26–28]. TiO_2_ and TiO_2_NP were treated twice as foliar spray with equal amounts (2000 mL) to run-off to foliage. The first application was applied after 60 days of sowing and the second was after 15 days from the first application (during vegetative stage). TiO_2_ and TiO_2_NP were prepared in 1L volumetric flask by dissolve 0.05 or 0.1 g pure TiO_2_ or TiO_2_NP in distilled water to bring the total solution volume to 1L. The recommendations made by Egypt's Ministry of Agriculture were followed in all agricultural operations. According to Jackson [31] and Cottenie [32], Table 1 displays the physical and chemical characteristics of the soil employed in this investigation. Prior studies performed on fennel seedlings were used to assess the amounts of SALIW and TiO_2_.

**Table 1:** Specifications of the experimental soil

Items	values
Sand (%)	29.7
Silt (%)	17.6
Clay (%)	52.7
pH (1: 2.5)	7.7
EC (dS m^−1^)	1.8
Organic matter (%)	1.7
CaCO_3_ (%)	2.4
Total N (%)	27.8
P (mg 100 g^−1^ Soil)	17.3
Soluble Cations (mg 100 g^−1^ Soil)	
K	22.2
Fe	26.7
Mn	11.9
Zn	5.8
Cu	16.3
Ca	54.1
Mg	9.6
Na	44.6
Soluble Anions (mg 100 g^−1^ Soil)	
HCO_3_	68.8
Cl	42.8
CO_3_	27.9
SO_4_	33.8
NO3	17.9

### Harvesting

Fresh herbs (g plant^−1^) were determined from each treatment during vegetative and flowering stages. Plant height (cm plant^−1^) and herb dry weights (g plant^−1^) were recorded during vegetative, flowering and fruiting stages. Fruit dry weights (g plant^−1^) were measured during the ripening stage (240 days from the sowing).

### Isolation of essential oil (EO)

From dried fruits in each treatment, three replicates of each treatment were made. Fifty grams of dried fruits from each replicate were hydro-distilled for three hours using Clevenger-style equipment [33]. Initial study suggests that hydro-distillation process should remain until no more EO could be extracted. For hydro-distillation, divided samples and 1L of water were introduced to a 2L round bottomed flask. EO boiling temperature was set to 100 °C for the purpose of extraction. EO was collected, cleaned of any traces of water using anhydrous sodium sulphate, and preserved at 4 °C in a sealed tube until use. Yield (g 100 plant^−1^) and relative percentage (w/w) of EO content were determined.

### Investigation of EO

Using a Shimadzu gas chromatography/mass spectrometric device (GC–MS), EO was investigated. Utilizing calibration curves generated using gas chromatography studies of common components; quantification was performed using an external standard approach that was developed. Retention index (RI), standard materials, and mass spectral data from the NIST/NBS and Wiley 275.l libraries were used to identify the individual components of EO [34].

### Analysis of POTSP

Using the techniques given by Anonymous [35], chlorophyll *a*, *b* and total carotenoids were quantified in fresh leaves {during vegetative stage (80 days from the sowing) and flowering stages (120 days from the sowing)} from each treatment. Fresh leaf tissues were pulverized in a mortar and pestle with 80% acetone. The optical density of the solution was measured using a spectrophotometer (Shimadzu UV-1700, Tokyo, Japan) at 662, 645 and 470 nm for Ch *a*, Ch *b* and carotenoids, respectively. Values for POTSP were given in mg g^−1^ fresh weight.

### Extraction and assessment of AOE

The technique for extracting AOE through vegetative and flowering stages was disclosed by Mukherjee [36]. The technique of Kar [37] was used to measure catalase activity (CAT), EC 1.11.1.6. Superoxide dismutase activity (SOD) EC 1.15.1.1 was identified by measuring inhibition of auto-oxidation of pyrogallol with the method of Marklund [38]. Peroxidase activity (POX) EC 1.11.1.7 assayed with the method of Kar [37] with slight modifications.

### TSOLS determination

TSOLS content of dried leaves collected from each treatment during vegetative and flowering stages was determined using the Dubois [39]. To create the extract, a foliar tissue (0.03 g) was homogenized with 80% ethanol and a standard D-glucose solution was used to quantify using absorbance measurements at 490 nm.

### Evaluation of TPHEN

According to Singleton [40], TPHEN content of leaf samples obtained at the vegetative and blooming phases was determined using the Folin-Ciocalteu reagent. In a nutshell, distilled water was used to dilute 2 mL of crude extract (1 mg mL^−1^) to 3 mL, 0.5 mL of Folin-Ciocalteu reagent must be well mixed with the sample for three minutes, and 2 mL of 20% (w/v) sodium carbonate were added after that. The mixture was permitted to stand for a further hour in complete darkness, as well as measuring absorbance at 650 nm. The TPHEN content was calculated using the calibration curve, and the findings were shown as mg of gallic acid equivalent per gram of dry weight.

### Estimation of TFLAV

According to Pourmorad [41], the aluminum chloride colorimetric technique was used to quantify the TFLAV in leaves taken throughout the vegetative and flowering periods. In a nutshell, 50 L of crude extract (1 mg mL^−1^ ethanol) were diluted with methanol to get 1 mL, 4 mL of distilled water was combined with 0.3 mL of a 5% NaNO_2_ solution; after incubating for five minutes, 0.3 mL of 10% AlCl_3_ solution was added, and the mixture was let to stand for six minutes. 2 mL of a 1 mol L^−1^ NaOH solution was then added, Double-distilled water was used to dilute the mixture to its final amount of 10 mL. The mixture was let to stand for 15 min, then, it was detected at 510 nm. TFLAV content was calculated using a calibration curve; it was then given as mg of rutin equivalent per g of dry weight.

### Determining the PROL

Using the technique described by Bates [42], PROL content of fresh leaves taken throughout the vegetative and blooming periods was measured. PROL extract, acid ninhydrin, and glacial acetic acid were all added, and they were then incubated for one hour in a boiling water bath and then an ice bath. Using a Spekol Spectrocololourimeter VEB Carl Zeiss, the absorbance was determined at 520 nm. Authentic PROL was used at a known concentration to create a standard curve.

### Measuring H_2_O_2_ and lipid peroxidation

Malondialdehyde (MDA) content was tested in order to determine the amount of lipid peroxidation using Heath's method [43]. A fresh leaf sample (collected at vegetative and flowering stages) weighing 0.5 g was homogenised in 10 ml of 5% tri-chloroacetic acid (TCA). Centrifuging the homogenate at 15,000 × g for 10 min. 2.0 ml of the supernatant was mixed with 4.0 ml of 0.5% thiobarbituric acid (TBA) in 20% TCA. The mixture was heated for 30 min at 95 °C, then immediately cooled in an ice bath and centrifuged for 10 min at 10,000 × g. using a JASCO V-750 spectrophotometer; the absorbance was measured at 500 nm and at 600 nm for non-specific absorption. A 155 mmol L^−1^ cm^−1^ extinction coefficient was used to compute the MDA concentration furthermore presented as nmol (MDA) g^−1^ FW.

The technique of Yu [44] was used to measure the H_2_O_2_ in the leaf samples (taken at vegetative and blooming phases). In a mortar that had already been cooled, 0.5 g of fresh leaf tissue and 5.0 ml of 0.1% (w/v) TCA were combined. After 15 min of centrifugation at 12,000 g, 0.5 ml of the supernatant was added to 1.0 ml of potassium iodide (KI) and 0.5 ml of potassium phosphate buffer (pH 7). Using a JASCO V-750 spectrophotometer, the absorbance was determined at 390 nm. Using the extinction value of 0.28 m^−1^ cm^−1^, the quantity of H_2_O_2_ was determined, and measured in nmol g^−1^ FW.

### Data evaluation

In this experiment, two variables were used: irrigation water sources (IRWS) such as FRIW & SALIW, and TiO_2_ (5 rates). There were three replicates with ten pots for each one. The experiment employed a block design that was entirely random. According to Snedecor [45], the average data from both seasons were statistically analyzed using two ways analysis of variance. Variations between means were assessed by least of significant differences (LSD) at 0.05. Pearson’s correlation was used to identify the relationships between growth characters and chemical variables during various growth stages. Also Pearson’s correlation was used to identify the relationships EO content and EO compositions. The interactions between IRW and TiO_2_ as well as the individual (mean or overall) impacts of each factor were investigated in the results. The STAT-ITCF software claims that this approach was used in such ways [46].

## Results

### Ti forms, IRWS, and their interactions have an impact on the morphological characters (MORC)

**Fig. 2:**
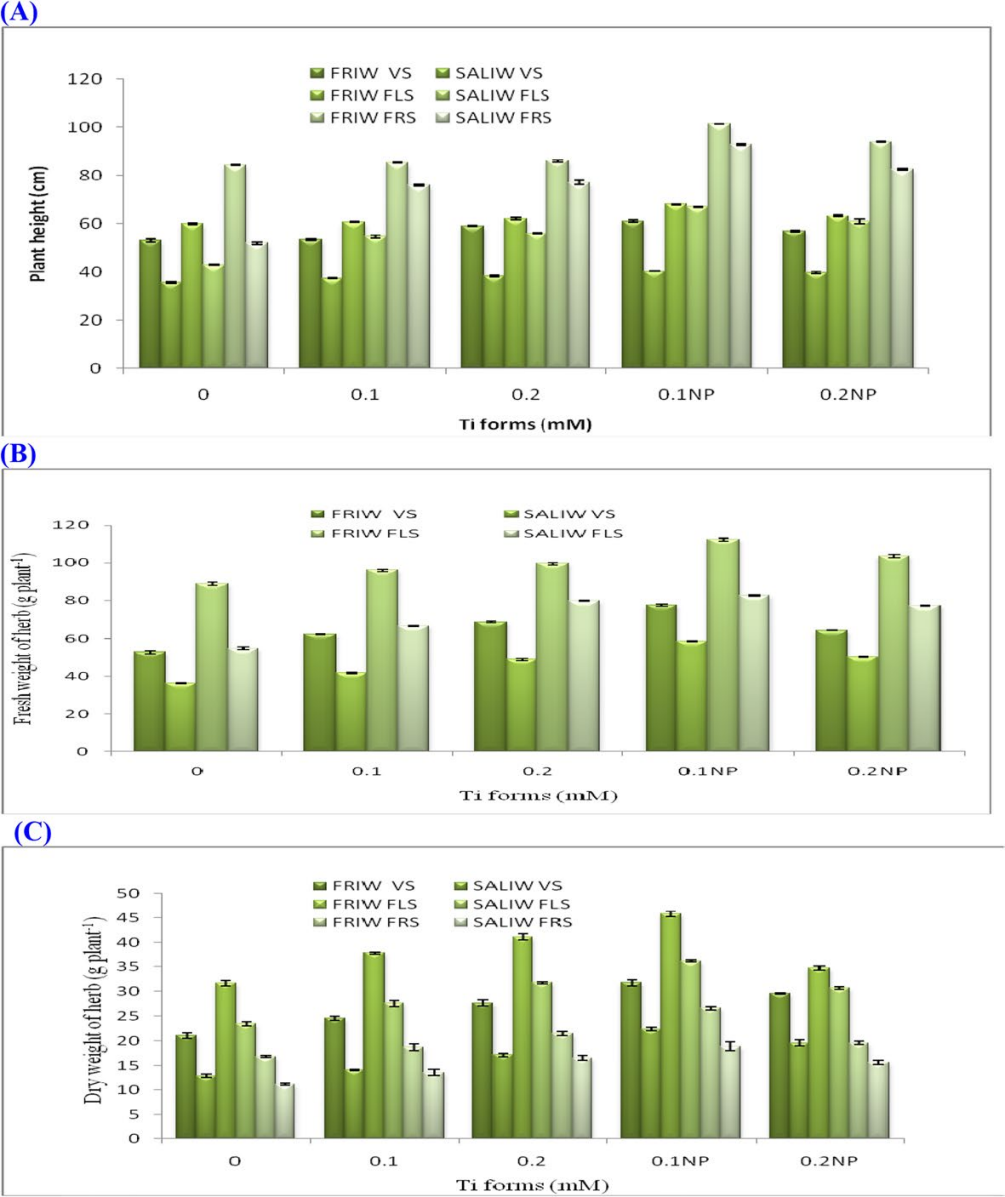
**A** Effect of Ti forms and different irrigation water sources (IRWS) on plant height. **B** Impact of Ti forms and IRWS on fresh weight mass production. **C** Impact of Ti forms and IRWS on dry weight mass production. FLS, flowering stage; FRIW, fresh irrigation water; FRS, fruiting stage; SALIW, salted irrigation water; VS, vegetative stage. Every value is shown as mean ± SD (standard divination)

**Fig. 3:**
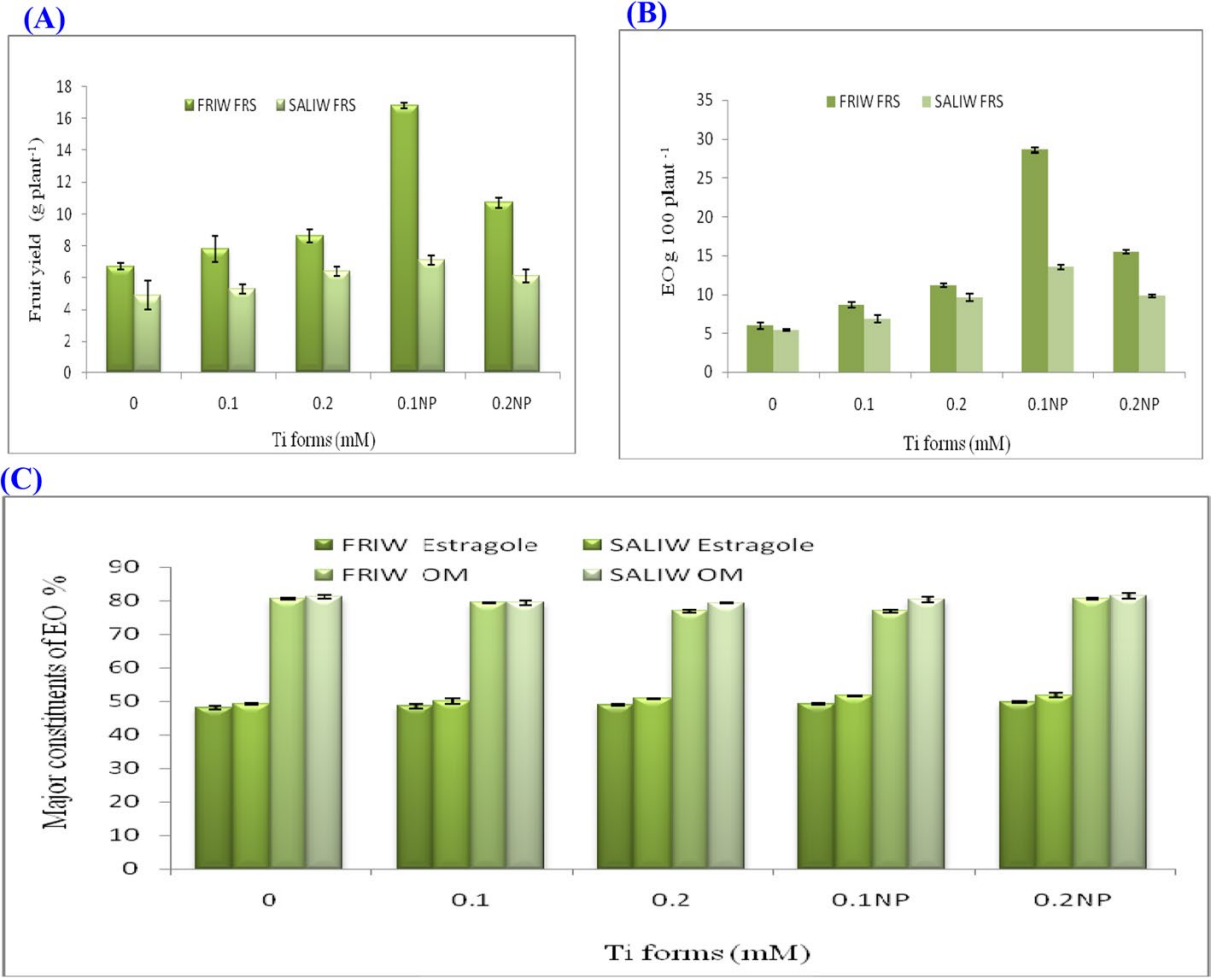
**A** Effect of Ti forms and irrigation water sources (IRWS) on fruit yield. **B** Ti forms and IRWS's effect on essential oil (EO) yield. **C** Ti forms and IRWS changed the EO's estragole and oxygenated monoterpenes (OM). FRIW, fresh irrigation water; FRS, fruiting stage; SALIW, salted irrigation water. All values are given as mean ± SD (standard divination)

Effects of TiO_2_ or TiO_2_NP, IRWS and their interactions on MORC such as plant height, fresh and dry mass production (g plant^−1^) and fruit yield (g plant^−1^) during different growth stages are shown in Table 2 and Figs. 2A-C and 3A). Changes in the rates of Ti forms and IRWS had a significant impact on MORC. Addition of SALIW resulted in a significant decrease in all MORC. Under varying amounts of Ti forms with IRWS, various MORC significantly enhanced. The maximum values of plant height (101.3 cm at fruiting stage) fresh mass (112.2 g plant^−1^ at flowering stage), dry mass (26.6 g plant^−1^ at flowering stage) and ripening fruit yield (16.8 g plant^−1^) were recorded when plants received 0.1 mM TiO_2_NP with FRIW.

**Table 2:** Effect of IRWS, Ti forms and their interactions on the MORC and EO content

IRWS	Ti forms (mM)	Plant height (cm)			Weight of herb (g plant^−1^)					Fruit yield	EO	
					Fresh		Dry					
		.cm			g plant^−1^						g 100 plant^−1^	%
		VS	FLS	FRS	VS	FLS	VS	FLS	FRS	FRS		
FRIW	0.0	53.0	60.0	84.3	52.8	88.9	21.1	31.7	16.8	6.7	6.0	0.9
	0.1	53.3	60.7	85.3	62.4	96.2	24.6	37.8	18.7	7.8	8.6	1.1
	0.2	59.0	62.0	86.0	68.8	99.7	27.7	41.1	21.5	8.6	11.2	1.3
	0.1NP	61.0	68.0	101.3	77.6	112.6	31.8	45.8	26.6	16.8	28.6	1.7
	0.2NP	57.0	63.3	94.0	64.4	103.7	29.6	34.8	19.6	10.7	15.5	1.4
Overall FRIW		56.7	62.8	90.2	65.2	100.2	27.0	38.2	20.6	10.1	14.0	1.3
SALIW	0.0	35.6	43.0	52.0	36.3	54.9	12.9	23.4	11.2	4.9	5.4	1.1
	0.1	37.3	54.7	76.0	41.7	66.8	14.1	27.6	13.6	5.3	6.9	1.3
	0.2	38.3	56.0	77.0	48.9	79.7	17.1	31.8	16.5	6.4	9.6	1.5
	0.1NP	40.3	67.0	92.7	58.6	82.6	22.4	36.2	18.9	7.1	13.5	1.9
	0.2NP	39.6	61.0	82.5	50.3	77.3	19.6	30.7	15.6	6.1	9.8	1.6
Overall SALIW		38.2	56.3	70.0	47.2	72.3	17.2	29.9	15.2	6.0	9.0	1.5
Overall Ti forms	0.0	44.3	51.5	68.2	44.6	71.9	17.0	27.6	14.0	5.8	5.7	1.0
	0.1	45.3	57.7	80.7	52.1	81.5	19.4	32.7	16.2	6.6	7.8	1.2
	0.2	48.7	59.0	81.5	58.9	89.7	22.4	36.5	19.0	7.5	10.4	1.4
	0.1NP	50.7	67.5	97.0	68.1	97.6	27.1	41.0	22.8	12.0	21.1	1.8
	0.2NP	48.3	62.2	88.3	57.4	90.5	24.6	32.8	17.6	8.4	12.7	1.5
LSD (0.05)												
IRWS		1.1	2.1	2.5	2.1	1.2	0.9	1.2	3.3	0.9	2.3	ns
Ti forms		3.4	3.2	3.9	2.5	2.3	1.1	1.6	5.1	1.1	3.1	0.1
IRWS x Ti forms		4.2	4.1	5.1	2.9	2.7	1.3	2.1	7.2	1.2	5.4	ns

### Ti form, IRWS and their interactions' effects on EO composition

Significant reductions in EO output (g 100 plants^−1^) were seen in response to SAIW compared with FRIW application (Table 2; Fig. 3B); however, it is evident that adding both Ti forms resulted in significant increases of EO yield. The greatest yield of EO (28.6 g 100 plants^−1^) was recorded with the treatment of 0.1 mM TiO_2_NP x FRIW (Table 2). According to Table (2), various increments in EO content have been observed under different IRWS, Ti forms and their interactions. Plants that received 0.1 mM TiO2NP x SALIW had the highest amount of EO content (1.9%). The increments of EO contents were significant for Ti forms, but they were non significant for IRWS and Ti forms x IRWS.

EO from fennel fruits contained 23 components under IRWS, Ti forms and their interactions (Tables 3 and 4; Fig. 3C). Estragole, limonene, carvacrol, and carvone were the main ingredients. Different recognized components were present in three chemical fractions; Monoterpene hydrocarbons (MH) and sesquiterpene hydrocarbons (SH) were the minor fractions, whereas oxygenated monoterpenes (OM) made up the majority. Significant variations were observed in the major components and all chemical groups as a result of IRWS, Ti forms, and their interactions. *Effect of IRWS x Ti forms* (Table 3): plants exposed to 0.2 mM TiO_2_NP x SALIW produced the maximum amounts of estragole (51.9%), limonene (12.9%), carvacrol (12.9%), carvone (12.9%) and OM (81.5%). The greatest value of OM (19.7%) was obtained from plants subjected to 0.1 mM TiO_2_NP x FRIW; while, plants treated with 0.2 mM TiO_2_ x FRIW gave the maximum value of SH (4.7%). *Effect of Ti forms* (Table 4): treatment of 0.2 mM TiO_2_NP gave the highest value of estragole (50.9%), limonene (12.8%), carvacrol (12.3%), carvone (12.0%) and OM (81.1%). The level of 0.1 mM TiO_2_NP0 resulted in the maximum value of MH (18.7%), while, untreated plants gave the highest amount of SH (3.0%). *Effect of IRWS***:** plants exposed to SALIW produced the highest amounts of estragole (50.7%), limonene (12.1%), carvacrol (11.6%), carvone (11.8%) and OM (80.3%); while those subjected to FRIW resulted in the highest values of MH (17.8%) and SH (3.1%). The statistical changes in various components and their groups in response to IRWS, Ti forms and their interactions were presented in Tables 3 and 4.

**Table 3:** IRWS x Ti forms' impact on the constituents of EO

No	Constituents (%)	RI	IRWS										LSD (0.05)
			FRIW					SALIW					
			Ti forms (mM)										
			0.0	0.1	0.2	0.1NP	0.2NP	0.0	0.1	0.2	0.1NP	0.2NP	
1	.α-Pinene	930	1.5	0.5	0.8	0.9	0.6	0.7	0.7	0.5	0.6	0.9	0.3
2	Camphene	953	0.5	0.8	0.8	0.6	0.6	0.4	0.5	0.4	0.7	0.4	0.2
3	.β-Pinene	980	0.7	0.9	0.7	0.9	0.5	0.5	1.1	0.8	0.2	0.3	0.5
4	Myrcene	991	0.4	1.4	1.6	1.4	0.4	0.6	0.5	0.7	0.7	0.7	0.4
5	.α-Phellandrene	1005	0.2	1.4	0.9	0.5	0.4	0.3	1.1	0.6	0.7	0.8	0.6
6	.*p*-cymene	1026	0.6	1.2	0.4	2.4	1.3	0.4	0.3	0.7	0.6	0.6	0.5
7	Limonene	1031	10.9	11.1	11.5	11.8	11.9	11.7	11.9	12.3	12.8	12.9	ns
8	*.trans*-β-Ocimene	1050	0.3	0.4	0.5	0.4	0.8	0.5	0.5	0.5	0.5	0.4	0.2
9	.γ-Terpinene	1062	0.2	1.1	1.1	0.8	0.5	1.3	1.6	0.5	0.4	0.7	0.7
10	Fenchone	1094	2.5	1.6	0.7	0.8	0.5	1.5	0.8	0.6	0.5	0.6	0.8
11	Terpinen-4-ol	1177	0.7	1.7	0.9	0.3	0.4	0.6	0.5	0.9	0.5	0.7	0.5
12	.α-Terpineol	1189	0.8	1.4	0.9	2.2	2.7	1.9	1.1	0.5	0.8	0.5	0.6
13	Estragole	1195	48.1	48.5	48.9	49.2	49.8	49.2	50.1	50.8	51.7	51.9	0.9
14	*.cis*-Carveol	1229	1.8	2.1	1.4	0.9	0.7	2.2	1.6	1.2	0.5	0.3	0.3
15	Pulegone	1237	2.9	2.8	1.6	0.7	0.5	1.1	1.5	0.9	0.6	0.4	0.7
16	Carvone	1242	9.7	9.9	10.4	10.5	11.1	10.5	11.4	11.9	12.5	12.9	0.8
17	Anethole	1255	1.5	0.8	0.8	0.5	0.9	1.7	0.7	0.3	0.4	0.8	0.9
18	Thymol	1290	2.8	0.5	0.5	0.8	2.5	2.2	0.8	0.7	0.6	0.5	0.7
19	Carvacrol	1298	9.9	10.1	10.7	10.9	11.6	10.3	10.9	11.5	12.2	12.9	0.9
20	Caryophyllene	1428	1.1	0.4	1.7	0.7	0.4	0.9	0.5	0.6	0.7	0.2	0.4
21	.β-Humulene	1440	1.1	0.7	1.6	0.5	0.3	0.4	0.4	1.6	0.6	0.6	0.4
22	Germacrene D	1490	0.9	0.3	0.9	0.7	0.5	0.5	0.7	0.7	0.6	0.2	0.2
23	.β-Bisabolene	1509	0.7	0.2	0.5	1.3	0.8	0.3	0.6	0.7	0.5	0.5	0.4
MH (1–9)			15.3	18.8	18.3	19.7	17.0	16.4	18.2	17.0	17.2	16.8	1.1
OM(10–19)			80.7	79.4	76.8	76.8	80.7	81.2	79.4	79.3	80.3	81.5	1.2
SH (20–23)			3.8	1.6	4.7	3.2	2.0	2.1	2.2	3.6	2.4	1.5	1.3
Overall			99.8	99.8	99.8	99.7	99.7	99.7	99.8	99.9	99.9	99.8

**Table 4:** The effect of Ti forms or IRWS on the components of EO

No	Constituents (%)	RI	IRWS			Ti forms (mM)					LSD
			FRIW	SALIW	LSD	0.0	0.1	0.2	0.1NP	0.2NP	
1	.α-Pinene	930	0.9	0.7	0.1	1.1	0.6	0.7	0.8	0.8	0.2
2	Camphene	953	0.7	0.5	0.1	0.5	0.7	0.6	0.7	0.5	0.1
3	.β-Pinene	980	0.7	0.6	0.2	0.6	1.0	0.8	0.6	0.4	0.4
4	Myrcene	991	1.0	0.6	0.3	0.5	1.0	1.2	1.1	0.6	0.3
5	.α-Phellandrene	1005	0.7	0.7	ns	0.3	1.3	0.8	0.6	0.6	0.4
6	.*p*-cymene	1026	1.2	0.5	0.3	0.5	0.8	0.6	1.5	1.0	0.6
7	Limonene	1031	11.4	12.1	0.3	11.3	11.5	11.9	12.3	12.8	0.3
8	*.trans*-β-Ocimene	1050	0.5	0.5	ns	0.4	0.5	0.5	0.5	0.6	ns
9	.γ-Terpinene	1062	0.7	0.9	0.1	0.8	1.4	0.8	0.6	0.6	0.3
10	Fenchone	1094	1.2	0.8	0.2	2.0	1.2	0.7	0.7	0.6	0.2
11	Terpinen-4-ol	1177	0.8	0.6	0.1	0.7	1.1	0.9	0.4	0.6	0.5
12	.α-Terpineol	1189	1.6	1.0	0.2	1.4	1.3	0.7	1.5	1.6	0.6
13	Estragole	1195	48.9	50.7	0.9	48.7	49.3	49.9	50.5	50.9	0.6
14	*.cis*-Carveol	1229	1.4	1.2	0.1	2.0	1.9	1.3	0.7	0.5	0.4
15	Pulegone	1237	1.7	0.9	0.3	2.0	2.2	1.3	0.7	0.5	0.6
16	Carvone	1242	10.3	11.8	0.8	10.1	10.7	11.2	11.5	12.0	0.8
17	Anethole	1255	0.9	0.8	ns	1.6	0.8	0.6	0.6	0.9	0.3
18	Thymol	1290	1.4	1.0	0.2	2.5	0.7	0.6	0.6	1.5	0.6
19	Carvacrol	1298	10.6	11.6	0.6	10.1	10.5	11.1	11.6	12.3	0.9
20	Caryophyllene	1428	0.9	0.6	0.2	1.0	0.5	1.2	0.7	0.3	0.2
21	.β-Humulene	1440	0.8	0.7	ns	0.8	0.6	1.5	0.6	0.5	0.7
22	Germacrene D	1490	0.7	0.5	0.1	0.7	0.5	0.8	0.7	0.4	0.1
23	.β-Bisabolene	1509	0.7	0.5	0.1	0.5	0.4	0.6	0.9	0.7	0.2
MH (1–9)			17.8	17.1	0.2	15.9	18.5	17.7	18.7	16.9	0.9
OM (10–19)			78.9	80.3	0.6	81.0	79.4	78.1	78.6	81.1	0.7
SH (20–23)			3.1	2.4	0.7	3.0	1.9	4.1	2.8	1.8	0.6
Overall			99.8	99.8		99.9	99.8	99.9	99.9	99.8	

### Effects of Ti forms, IRWS and their interactions on the POTSP

**Fig. 4:**
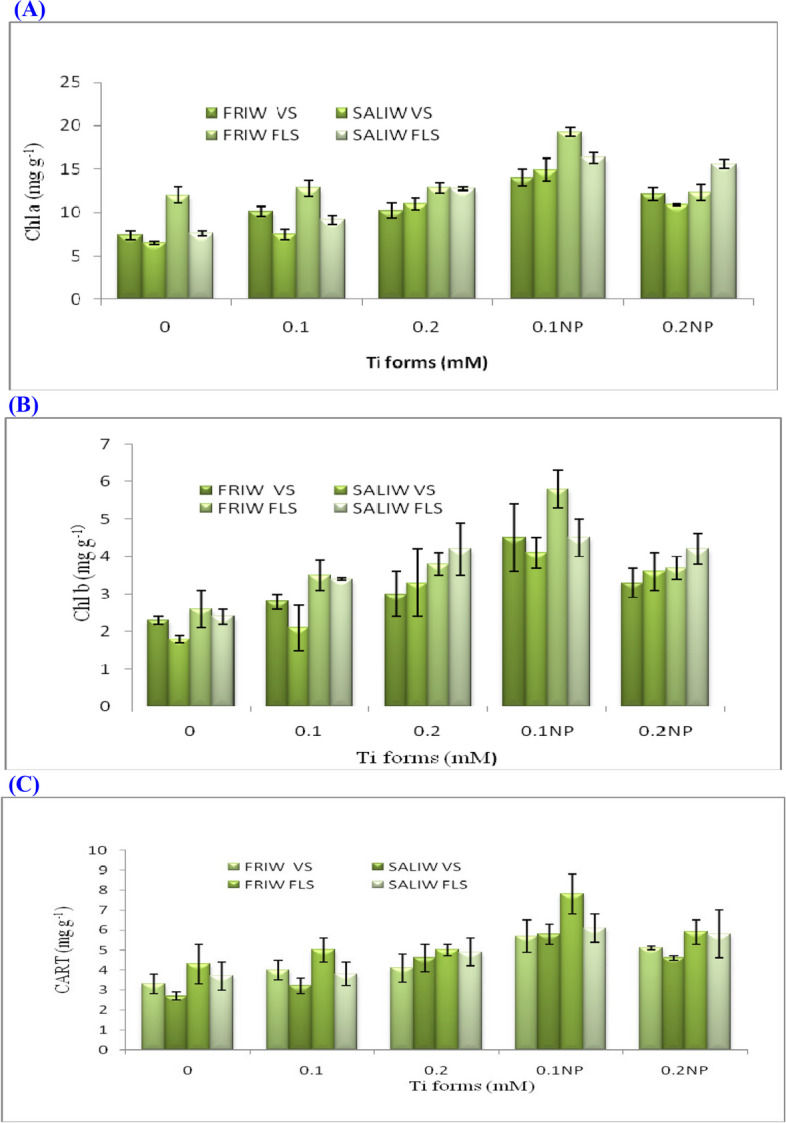
**A** Effect of Ti forms and irrigation water sources (IRWS) on chlorophyll *a* (Chl* a*). **B** Ti forms and IRWS's impact on Chl *b*. **C** Impact of IRWS and Ti forms on carotenoids (CART). FLS, flowering stage; FRIW, fresh irrigation water; SALIW, salted irrigation water; VS, vegetative stage. Each value is displayed as mean ± SD (standard divination)

During vegetative and blooming stages, SALIW led to a deficiency of POTSP (chlorophyll *a, b*, and total carotenoids); however, they were enhanced by applying both types of TiO_2_ at different development phases (Table 5; Fig. 4A-C). The maximum values of chlorophyll *a* (19.3 mg g^−1^) chlorophyll *b* (5.8 mg g^−1^) and total carotenoids (7.8 mg g^−1^) were obtained from the plant exposed to 0.1 mM TiO_2_NP x FRIW during the flowering stage. The variations in chlorophyll *a* were significant for IRWS, Ti forms and IRWS x Ti forms. The changes in chlorophyll *b* and total carotenoids were highly significant for Ti forms; while they were not significant for IRWS or IRWS x Ti forms (Table 5).

**Table 5:** POTSP and AOEA are impacted by IRWS x Ti forms

IRWS	Ti forms (mM)	POTSP (mg g^−1^)						AOEA (unit/g FW. min)					
		Chlorophyll *a*		Chlorophyll *b*		Carotenoids		POX		CAT		SOD	
		VS	FLS	VS	FLS	VS	FLS	VS	FLS	VS	FLS	VS	FLS
FRIW	0.0	7.4	12.0	2.3	2.6	3.3	4.3	3.4	5.6	1.7	8.3	1.4	2.8
	0.1	10.1	12.8	2.8	3.5	4.0	5.0	3.7	5.8	2.4	9.1	1.5	2.9
	0.2	10.2	12.8	3.0	3.8	4.1	5.0	4.0	6.4	3.3	9.8	1.7	3.0
	0.1NP	14.0	19.3	4.5	5.8	5.7	7.8	4.1	6.6	4.7	10.5	1.9	3.1
	0.2NP	12.1	12.3	3.3	3.7	5.1	5.9	4.6	6.7	4.1	10.2	2.0	3.2
Overall FRIW		10.8	13.8	3.2	3.9	4.4	5.6	4.0	6.2	3.3	9.6	1.7	3.0
SALIW	0.0	6.5	7.6	1.8	2.4	2.7	3.7	4.7	7.4	5.5	10.8	2.1	3.3
	0.1	7.5	9.1	2.1	3.4	3.2	3.8	4.8	8.0	5.9	11.2	2.1	3.5
	0.2	11.0	12.7	3.3	4.2	4.6	4.9	5.1	8.5	6.7	11.6	2.3	3.7
	0.1NP	14.9	16.3	4.1	4.5	5.8	6.1	5.2	10.5	7.9	13.6	2.6	4.0
	0.2NP	10.9	15.6	3.6	4.2	4.6	5.8	5.3	12.1	7.2	12.1	2.4	4.6
Overall SALIW		10.2	12.3	3.0	3.7	4.2	4.9	5.0	9.3	6.6	11.8	2.3	3.8
Overall Ti forms	0.0	7.0	9.8	2.1	2.5	3.0	4.0	4.1	6.5	3.6	9.5	1.7	3.1
	0.1	8.8	11.0	2.5	3.5	3.6	4.4	4.3	6.9	4.2	10.2	1.8	3.2
	0.2	10.6	12.8	3.2	4.0	4.4	5.0	4.6	7.4	4.9	10.7	2.0	3.4
	0.1NP	14.5	17.8	4.3	5.2	5.8	7.0	4.7	8.5	6.3	12.0	2.3	3.6
	0.2NP	11.5	14.0	3.5	4.0	4.9	5.9	5.0	9.4	5.6	11.1	2.2	3.9
LSD (0.05)													
IRWS		0.2	0.1	ns	ns	ns	ns	0.1	0.1	0.1	0.4	0.1	0.2
Ti forms		0.4	0.5	0.1	0.1	0.1	0.2	0.1	0.1	0.4	0.7	0.1	0.4
IRWS x Ti forms		0.5	0.8	ns	ns	ns	ns	0.2	0.3	0.5	1.1	0.2	0.7

### Effects of Ti forms, IRWS, and their interactions on the AOEA

**Fig. 5:**
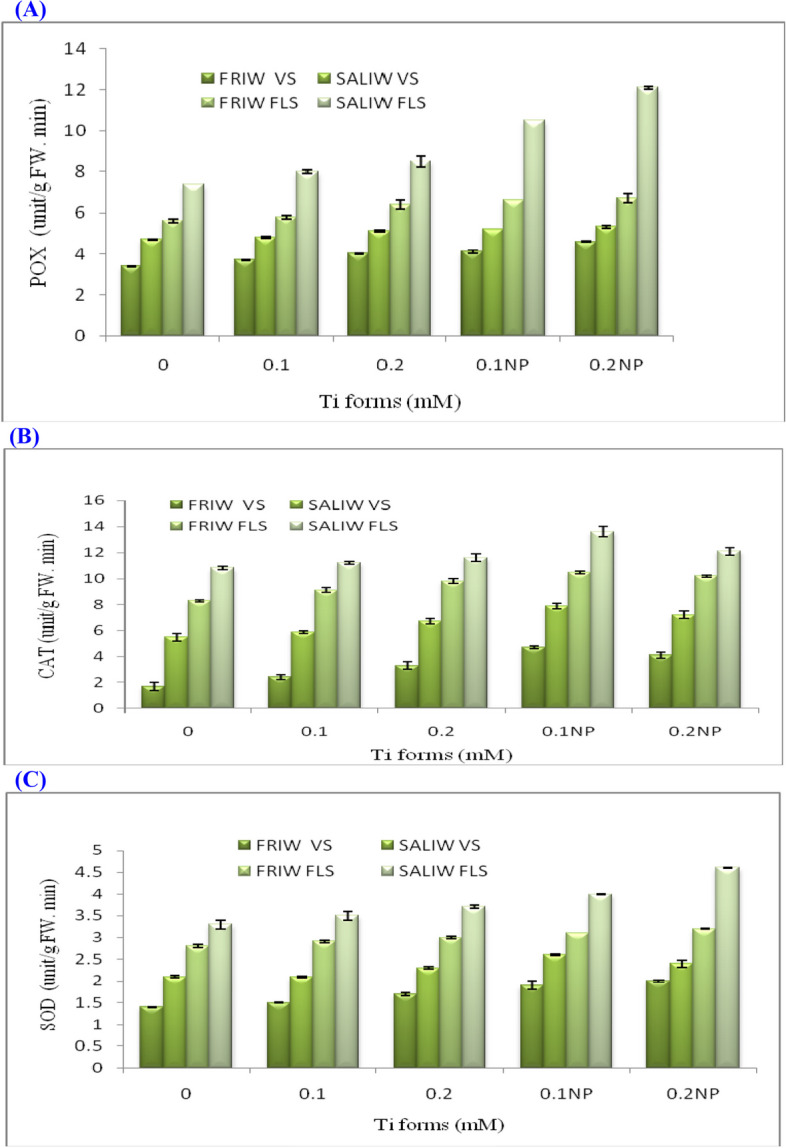
**A** Effect of Ti forms and irrigation water sources (IRWS) on peroxidase (POX). **B** Impact of IRWS and Ti forms on catalase (CAT). **C** Response of superoxide dismutase (SOD) to IRWS and Ti forms. FLS, flowering stage; FRIW, fresh irrigation water; SALIW, salted irrigation water; VS, vegetative stage. Every value is shown as mean ± SD (standard divination)

AOEA (POX, CAT and SOD) in plant cells were significantly increased during vegetative and flowering stages when plants were exposed to various IRWS and Ti forms (Table 5; Fig. 5A-C). Plants exposed to SALIW x Ti forms displayed greater levels of AOEA than those exposed to FRIW. The maximum values of POX (12.1 unit/g FW. min), CAT (12.1 unit/g FW. min) and SOD (4.6 unit/g FW. min) were resulted from plant treated with SALIW × 0.1 mM TiO_2_NP at flowering stage. The increments of POX, CAT and SOD were significant for IRWS, Ti forms and the interactions (Table 5).

### Ti forms, IRWS, and their interactions' effects on the TSOLS

**Fig. 6:**
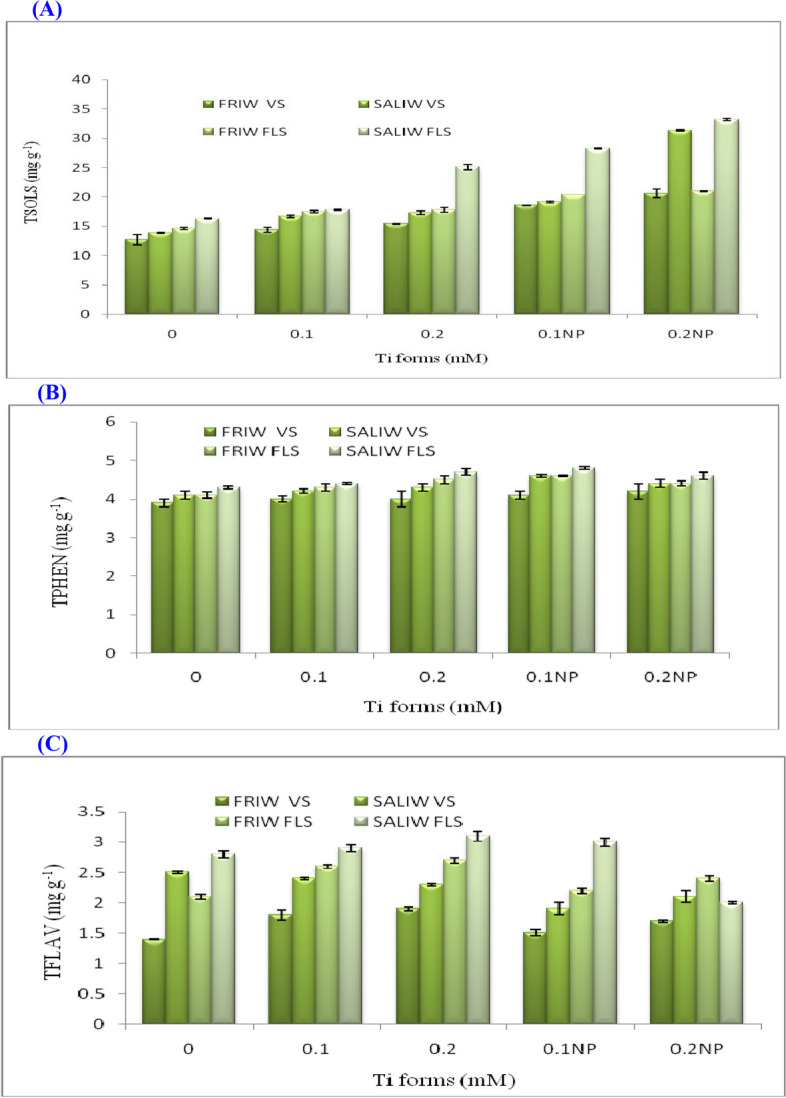
**A** Impact of Ti forms and irrigation water sources (IRWS) on total soluble sugars (TSOLS). **B** Ti forms and IRWS effect on total phenols (TPHEN). **C** Total flavonoids (TFLAV) are affected by Ti forms and IRWS. FLS, flowering stage; FRIW, fresh irrigation water; SALIW, salted irrigation water; VS, vegetative stage. All values are shown as mean ± SD (standard divination)

The contents of TSOLS significantly increased with various IRWS, TiO_2_ from and TiO_2_ from × IRWS at vegetative and flowering stages (Table 6; Fig. 6A). Plants treated with Ti forms x SALIW gave higher values in TSOLS than those exposed to Ti forms x FRIW. However, the highest value of TSOLS (33.2 mg g^−1^) resulted from 0.2 mM TiO_2_NP x SALIW during the flowering stage.

**Table 6:** IRWS x Ti forms' effects on the concentrations of TSOLS, TPHEN, TFLAV, PROL, H2O2, and MDA

IRWS	Ti forms (Mm)	TSOLS		TPHEN		TFLAV		PROL (µmoles g^−1^)		H_2_O_2_ (µM)		MDA (nmol g^−1^)	
		(mg g^−1^)											
		VS	FLS	VS	FLS	VS	FLS	VS	FLS	VS	FLS	VS	FLS
FRIW	0.0	12.7	14.6	3.9	4.1	1.4	2.1	1.2	2.2	10.4	11.8	6.3	22.1
	0.1	14.4	17.5	4.0	4.3	1.8	2.6	3.8	4.5	5.3	8.2	5.9	15.7
	0.2	15.4	17.8	4.0	4.5	1.9	2.7	4.3	5.0	5.0	8.1	3.8	14.5
	0.1NP	18.6	20.4	4.1	4.6	1.5	2.2	1.7	2.3	4.5	6.5	1.4	9.2
	0.2NP	20.6	21.0	4.2	4.4	1.7	2.4	3.2	3.4	2.9	3.5	4.4	15.6
Overall FRIW		16.3	18.3	4.0	4.4	1.7	2.4	2.8	3.5	5.6	7.6	4.4	15.4
SALIW	0.0	13.9	16.3	4.1	4.3	2.5	2.8	5.2	6.5	14.6	15.5	9.6	25.0
	0.1	16.7	17.8	4.2	4.4	2.4	2.9	6.5	11.5	9.5	9.9	6.4	15.6
	0.2	17.3	25.1	4.3	4.7	2.3	3.1	6.6	13.8	8.9	9.6	4.4	15.5
	0.1NP	19.1	28.2	4.6	4.8	1.9	3.0	6.7	15.7	5.2	7.8	1.5	12.5
	0.2NP	31.3	33.2	4.4	4.6	2.1	2.0	6.3	19.1	5.5	7.3	2.6	13.4
Overall SALIW		19.7	24.1	4.3	4.6	2.2	2.8	6.3	13.3	8.7	10.2	4.9	16.4
Overall Ti forms	0.0	13.3	15.5	4.0	4.2	2.0	2.5	3.2	4.4	12.5	13.7	8.0	23.6
	0.1	15.6	17.7	4.1	4.4	2.1	2.8	5.2	8.0	7.4	9.1	6.2	15.7
	0.2	16.4	21.5	4.2	4.6	2.1	2.9	5.5	9.4	7.0	8.9	4.1	15.0
	0.1NP	18.9	24.3	4.4	4.7	1.7	2.6	4.2	9.0	4.9	7.2	1.5	10.9
	0.2NP	26.0	27.1	4.3	4.5	1.9	2.2	4.8	11.3	4.2	5.4	3.5	14.5
LSD (0.05)													
IRWS		0.2	0.3	ns	0.1	0.1	0.2	0.1	0.9	1.1	1.9	0.2	0.9
Ti forms		0.7	0.7	ns	0.1	0.2	0.4	0.3	1.3	2.6	3.1	0.7	1.2
IRWS x Ti forms		1.9	1.1	0.6	0.2	0.3	0.8	0.9	1.7	4.4	4.2	1.1	1.7

### Effects of Ti forms, IRWS, and their interactions on TPHEN

Through both vegetative and flowering phases, application of various IRWS, Ti forms, and their interactions promoted the assembly of TPHEN in fennel plants (Table 6; Fig. 6B). Plants treated with Ti forms x SALIW produced more TPHEN than plants subjected to FRIW. The highest amount of TPHEN (4.8 mg g^−1^) was found in SALIW with 0.1 mM TiO_2_NP treatment. At vegetative stage, the increases in TPHEN were non significant for IRWS or Ti forms treatments, but they were significant for the interaction treatments (Table 6); on the other hand, they were significant for all treatments during flowering stage.

### Ti form, IRWS, and their interactions' effects on the TFLAV

Changes in the IRWS resulted in a significant increment of the TFLAV, either Ti forms or IRW x Ti forms produced several changes in the TFLAV contents during vegetative and flowering stages (Table 6; Fig. 6C). Plants treated with 0 mM TiO_2_ x SALIW at flowering stage produced the greatest amount of TFLAV with the value of 3.1 mg g^−1^.

### PROL rates and the effects of Ti forms, IRWS and their interactions

**Fig. 7:**
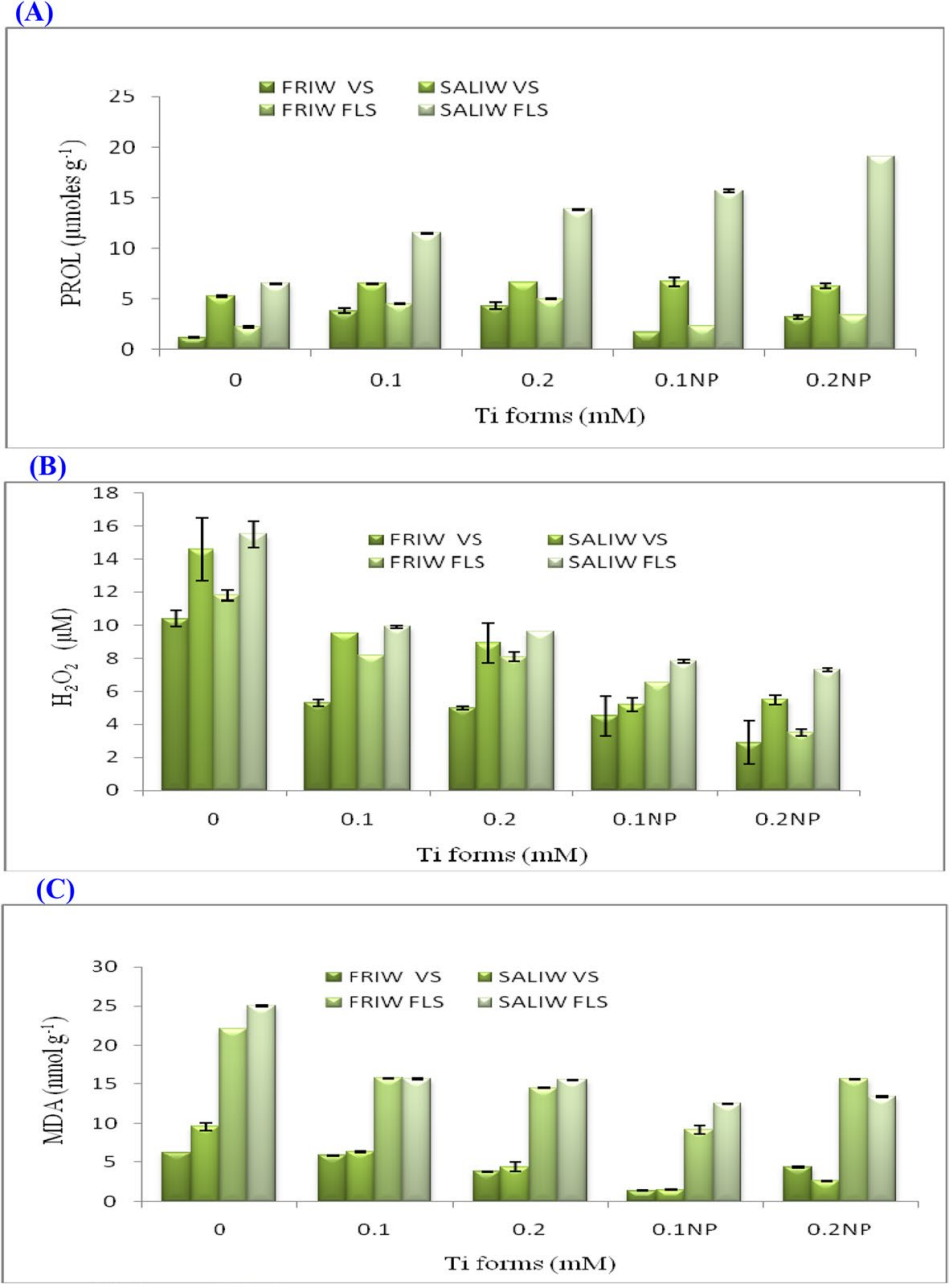
**A** Impact of irrigation water sources (IRWS) and Ti forms on proline (PROL). **B** Ti forms and IRWS's effect on hydrogen peroxide (H_2_O_2_). **C** Ti forms & IRWS and their impact on malondialdehyde (MDA). FLS, flowering stage; FRIW, fresh irrigation water; SALIW, salted irrigation water; VS, vegetative stage. All values are displayed as mean ± SD (standard divination)

The accumulations of PROL were significantly promoted by applying various IRWS, Ti forms and IRWS x Ti forms during vegetative and flowering stages (Table 6; Fig. 7A). Plants exposed to SALIW x Ti forms gave higher values in PROL concentrations than those subjected to FRIW x Ti forms during both stages. The maximum value of PROL (19.1 µmoles g^−1^) was recorded with plants treated with SALIW × 0.1 mM TiO_2_NP at flowering stage.

### Effects of Ti forms, IRWS, and their interactions on the H_2_O_2_ levels

As SALIW, significant increments in H_2_O_2_ accumulations were observed at vegetative and flowering stages compared with FRIW application; however, by using Ti forms, they were reduced (Table 6; Fig. 7B). Plants exposed to SALIW without Ti forms throughout the flowering stage had the highest H_2_O_2_ level (15.5 µM).

### Effects of Ti forms, IRWS, and their interactions on the MDA levels

Throughout both vegetative and flowering phases, application of various IRWS, Ti forms and their interactions promoted the assembly of MDA in fennel leaves (Table 6; Fig. 7C). Compared to plants exposed to IRWS in both phases, those exposed to IRWS x Ti forms produced lower concentrations of MDA. The peak MDA rate was discovered in SALIW without Ti forms (25 nmol g^−1^) during the blooming stage. The variations in MDA rates for IRWS, Ti forms and their interactions were significant (Table 6).

### Pearson’s correlation

**Fig. 8:**
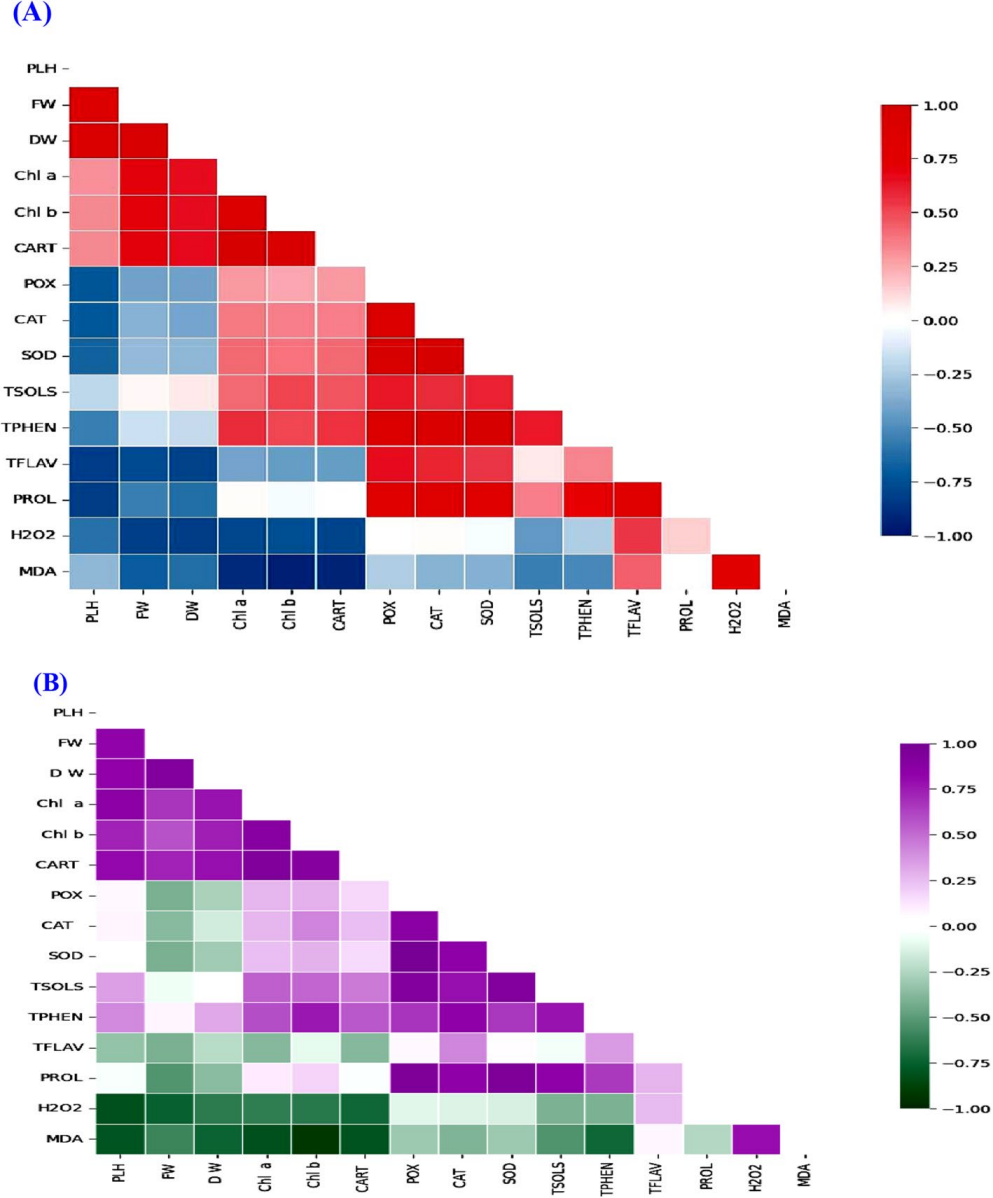
**A** Pearson’s correlation between morphological characters (MORC) and chemical contents variables in response to Ti forms and irrigation water sources (IRWS) during vegetative stage (VS). **B** Pearson’s correlation between MORC and chemical contents variables in response to Ti forms and IRWS during flowering stage (FLS). CART, carotenoids; CAT, catalase; Chl, chlorophyll; DW, dry weight; FW, fresh weight; H_2_O_2_, hydrogen peroxide MDA, malondialdehyde; PLH, plant height; POX, peroxidase; PROL, proline; SOD, superoxide dismutase; TFLAV, total flavonoids; TPHEN, total phenols; TSOLS, total soluble sugars

**Fig. 9:**
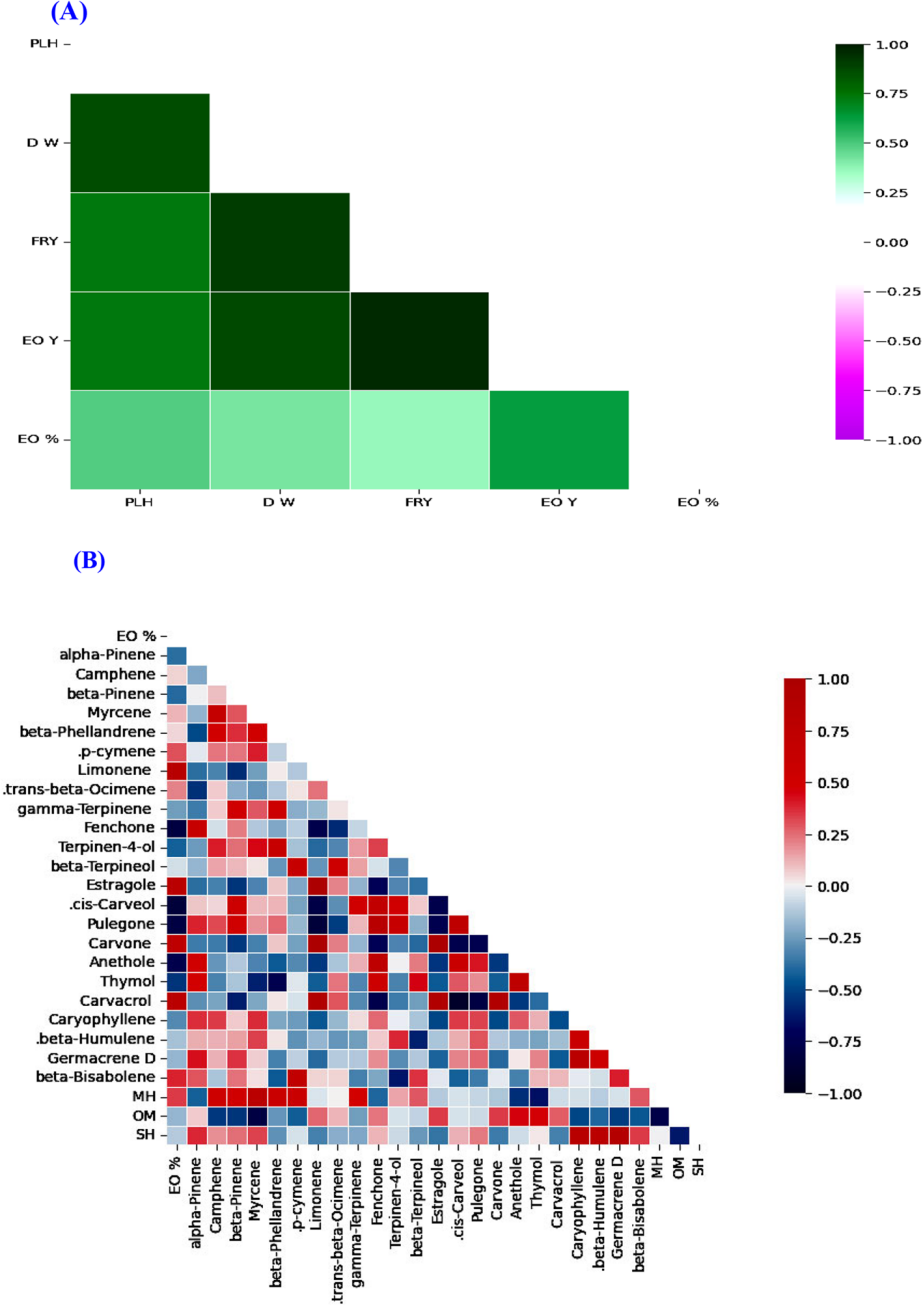
**A** Pearson’s correlation between morphological characters (MORC) and essential oil (EO) variables in response to Ti forms and irrigation water sources (IRWS) during fruiting stage (FRS). **B** Pearson’s correlation between EO (%) and EO constituent’s variables in response to Ti forms and IRWS. DW, dry weight; EO, essential oil; EOY, essential oil yield; FRY, fruit yield; MH, monoterpene hydrocarbons; OM, oxygenated monoterpenes; PLH, plant height; SH, sesquiterpene hydrocarbons

Pearson’s correlation was applied to analyze the relationship between MORC and chemical component in VS, FLS and FRS. Pearson’s correlation results exhibited positive and negative correlations between different variables. During VS and FLS (Fig. 8A and B), MORC variables and chemical component gave a strong correlation; highly strong correlation was found between PLH and FW or DW; FW and POTSP; AOEA and TPHEN. While low correlations were recorded between MDA, H_2_O_2_, PROL and AOEA. Urging FRS, PLH was strongly correlated with DW, fruit production and EO %; while low correlated with EO yield (Fig. 9A). Different correlations were noticed between EO constituents (Fig. 9B). Estragole had highly strong positive correlation with carvone and carvacrol; but strong negative correlation with *cis*-carveol and pulegone. Low correlation was obtained between limonene, *trans*-β-ocimene with β-bsabolene.

## Discussion

This research found that fennel plants exposed to SALIW experienced substantial reductions in plant height, fresh and dry weights and fruit yield; it might be as a result of SALIW, a source of toxic ions that raise the osmotic pressure of soil solution [47]; therefore, plant roots are unable to extract water from the surrounding soil, and plant exposure to drought causes a drop in turgor, which causes a decrease in plant cell development, which is followed by growth inhibition [48]. Fennel, which is known to be susceptible to abiotic stress factors [21], may be made to tolerate SALIW stress better by applying Ti forms to its leaves. Prior research has demonstrated beneficial effects of TiO_2_ form treatments (as foliar a spray) on the balance and absorption of critical macro and micronutrients; this causes chlorophyll and protein levels to rise, resulting in an increase of growth, dry matter content and yield under SALIW conditions [23]. However, the negative impact of SALIW on fennel's growth characteristics was overcome by the application of TiO_2_NP; possible causes include: plants treated with TiO_2_NP showed improved activities of AOE and enhanced accumulation of osmolytes. According to research, AOE are plants' first line of defense against ROS, and enhanced AOEA can change how well plants tolerate stress [49]. Furthermore, osmotic stress caused by SALIW was balanced by an increase in osmolytes, which provided the plant cells with improved osmotic pressure, allowing them to absorb more water as seen by an increase in leaf relative water content [50].

According to recent research, SALIW treatments reduce the production of POTSP; that since chloroplasts are degrading; this results in a lower buildup of carotenoids, chlorophyll *a*, and *b* [51]. However, plants exposed to Ti forms x SALIW accumulated more POTSP than plants exposed to SALIW. The potential for valence shift makes the TiO_2_ form unique among the transition forms; as a result, it takes part in activities involving the transfer of electrons related to photosynthesis [52]. Due to the development of a Ti-ascorbate compound in recent years, which can be applied as a foliar spray to plants, the usage of TiO_2_ in various crops has been reported in certain research studies to have significantly boosted photosynthesis [52]. The rates of POTSP were accelerated by foliar application of TiO_2_NP x SALIW [53]; this could be as a result of TiO_2_NP's ability to increase light absorption, speed up the transport and conversion of light energy, shield chloroplasts from ageing, and extend the duration during which they are able to perform photosynthetic activity [54].

In response to the applications of SALIW and Ti forms, several modifications were seen in EOs and their components. Numerous alterations to EOs and their constituents occur when fennel plants are exposed to SALIW or SALIW x Ti forms; these modifications can be attributed to differences in the enzymatic activity of EO formations [10]. EO is thought to increase an aromatic plant's tolerance to unfavorable circumstances like SALIW [10]. The elevation in EO (%) in response to SALIW with Ti forms may be explained by an increase in the number of glandular hairs as well as a corresponding increase in their densities [55]; while the differences in plant dry matter when exposed to SALIW or SALIW x Ti forms can be linked to the changes in EO yield [55, 56].

Treatments with SALIW resulted in an increase in the buildup of TSOLS; the storage of TSOLS for long-term energy supply, prolonged metabolism, and improved plant recovery may be the cause of the rise in TSOLS under SALIW [57]. In addition, SALIW x Ti forms treated plants showed greater accumulation TSOLS than SALIW-treated plants. Higher photosynthetic activity was closely correlated with a greater buildup of TSOLS in leaves in plants treated with SALIW x Ti forms [58]. Lack of study has made it difficult to currently understand the relationship between SALIW and the accumulation of antioxidant molecules.

Plants increase their TPHEN and TFLAV production in response to SALIW, which, following their role as antioxidants in preventing oxidative damage, provide a twofold protective impact, as the health-improving substances found in food plants [59]. Because TPHEN and TFLAV have the ability to free radical scavenging (FRRS) that kills plant cells, increasing the antioxidant capabilities are correlated with antioxidants substances levels (polyphenols and TFLAV) [60, 61]. Plants treated with SALIW x Ti forms gave higher amounts of TPHEN and TFLAV than those exposed to SALIW; these results confirmed by Ghorbanpour [62].

SALIW advocated for the accumulation of PROL in fennel leaves. These findings are consistent with the findings of Blum [63], as well as Slama [57] who revealed that PROL is regarded a source of energy, carbon, and nitrogen for regenerating plant tissues under SALIW. PROL content of fennel plants treated with Ti forms as a foliar spray x SALIW was higher than when treated with SALIW, these findings are confirmed by Lima [64].

The rise in H_2_O_2_ content in response to SALIW might be attributed to oxidative stress induced by SALIW, as well as peroxidation of membrane lipids, as represented by the higher concentration of H_2_O_2_; on the contrary, foliar spray Ti forms to salt-stressed plants lowered the amount of oxidative stress and lipid peroxidation, as evidenced by a decrease in H_2_O_2_ concentration [65].

SALIW causes an increase in MDA content in plant tissues as a lipid peroxidation product, which shows the oxidative damage induced by SALIW, leading to membrane damage; an increase in MDA level causes membrane damage and subsequent leaking of membrane electrolytes [66]. Mustafa [67] validated these findings that Ti forms inhibit MDA generation.

There are some earlier research publications on various plants that corroborate our conclusions on the usage of Ti forms in increasing plant development and yield under SALIW. TiO_2_ may act as an elicitor to improve secondary metabolism in sage plants for biosynthesis of natural antioxidants (TPHEN and TFLAV), EO and its main constituents (camphene, *p*-cymene, 1, 8-cineol, *cis*-thujene and camphor) [62]. It was discovered that using TiO_2_NP as a spray under abiotic stress factors had a significant influence on thyme plant growth characteristics and EO compositions [55]. TiO_2_NP improved tomato plant growth, yield, and quality under SALIW by increasing the rates of carbonic anhydrate, nitrate reductase, AOEA, PROL, and Glycine Betaine [65]. TiO_2_ x SALIW treatments increased tomato yield parameters; while they reduced pH value, TA, EC, TSS and TSS/TA ratio in the fruits [27]. TiO_2_ or TiO_2_NP applied via leaves at low concentrations has been documented to improve crop performance through stimulating the activity of certain enzymes, enhancing chlorophyll content and photosynthesis, promoting nutrient uptake, strengthening stress tolerance and improving crop yield and quality [25]. TiO_2_NP application on Moldavian balm cultivated under SALIW enhanced agronomic characteristics, EO yield, geranial, citral, geranyl acetate, geraniol, and AOEA; however, it decreased H_2_O_2_ production [28]. TiO_2_NP increased wheat germination properties, yield features, osmotic, water potential, carotenoids, TPHEN, TFLAV, TSOLS, proteins, PROL, amino acid contents and AOEA; whereas it lowered MDA at various SALIW doses; as a result, foliar application of TiO_2_NP can aid in plant resistance to SALIW [67]. TiO_2_ have a positive effect on the yield, macro & micronutrients and antioxidant activity of tomato plants under SALIW [68]. Several farmers would benefit from this experiment since they will be able to produce fennel plant in Egypt's new territories, which would reduce the negative impacts of SALIW and boost the chances of exporting fennel to other countries. Furthermore, it has been stated that the creation of fennel EO would allegedly require Ti application, as they greatly affect its synthesis, resulting in the extension of its biological domain as a natural source of EO.

## Conclusion

TiO_2_ forms increased production of fennel plants exposed to SALIW. Ti responded to SALIW by increasing POTSP, PROL, carbohydrates, EO, and antioxidants while lowering H_2_O_2_ and MDA. Application of TiO2NP is proposed as a viable strategy to lessen the detrimental effects of salinized irrigation water on fennel plant.

## Data Availability

The corresponding author can provide the datasets used and/or analyzed during the current work upon proper request.

## Abbreviations

AlCl_3_: Aluminum chloride
AOEA: Antioxidant enzymes activity
Ca: Calcium
CAT: Catalase
Chl: Chlorophyll
CART: Carotenoids
DW: Dry weight
DLS: Dynamic light scattering
dS m: Deci siemens per metre
EC: Electrical conductivity
EO: Essential oil
Fig: Figure
FLS: Flowering stage
FRIW: Fresh irrigation water
FRRS: Free radical scavenging
FRS: Fruiting stage
FW: Fresh weight
FWC: Field water capacity
GC/MS: Gas chromatography/mass spectrometric
H_2_O_2_: Hydrogen peroxide
IRWS: Irrigation water sources
K: Potassium
L: Liter
LSD: Least of significant differences
min: Minute
MDA: Malondialdehyde
Mg: Magnesium
MH: Monoterpene hydrocarbons
mM: milli Molar
MORC: Morphological characters
Na: Sodium
NaCl: Sodium chloride
NaNO_2_: Sodium nitrite
NP: Nano particles
ns: non significant
OM: Oxygenated monoterpenes
PLH: Plant height
POTSP: Photosynthetic pigments
POX: Peroxidase activity
PROL: Proline
PROT: Proteins
RI: Retention index
ROS: Reactive oxygen species
SALIW: Salted irrigation water
SD: Standard divination
SH: Sesquiterpene hydrocarbons
SOD: Superoxide dismutase
TA: Titratable acidity
TEM: Transmission electron microscope
TCARB: Total carbohydrates
TFLAV: Total flavonoids
Ti: Titanium
TiO_2_: Titanium dioxide
TiO_2_NP: Titanium dioxide nano particles
TPHEN: Total phenols
TSOLS: Total soluble sugars
TSS: Total soluble solids
VS: Vegetative stage
XRD: X-ray diffraction
